# Treatment of patellar dislocation with arthroscopic medial patellofemoral ligament reconstruction using gracilis tendon autograft and modified double-patellar tunnel technique: minimum 5-year patient-reported outcomes

**DOI:** 10.1186/s13018-020-1556-4

**Published:** 2020-01-22

**Authors:** Guanying Gao, Ping Liu, Yan Xu

**Affiliations:** Institute of Sports Medicine, Peking University Third Hospital, 49 North Garden Road, Haidian District, Beijing, 100191 China

**Keywords:** Medial patellofemoral ligament, Reconstruction, Recurrent patellar dislocation, Patient-reported outcomes

## Abstract

**Background:**

The purpose of this study was to retrospectively analyze the clinical outcomes of patients with recurrent patellar dislocation who underwent arthroscopic medial patellofemoral ligament (MPFL) reconstruction using gracilis tendon autograft and a modified double-patellar tunnel method. We hypothesized that our modified method would provide good clinical outcomes.

**Methods:**

Patients who underwent arthroscopic MPFL reconstruction with autograft gracilis tendon and modified double-patellar tunnels technique for recurrent patellar dislocation and were followed up for a minimum of 5 years were identified, and the clinical and follow-up data were retrospectively analyzed. Preoperatively, joint hypermobility was assessed with the Beighton score. The Insall–Salvati ratio, TT–TG distance, and Q angle were measured on radiographic images. Patient-reported outcomes including the Kujala, Lysholm, and Tegner scores were collected preoperatively and postoperatively. Patient satisfaction was assessed at the end of 5 years. Complications and recurrent dislocation occurring after surgery were recorded.

**Results:**

A total of 79 patients (94 knees) were enrolled; of these, 13 (16.5%) were lost to follow-up. The data of 66 patients (80 knees) were available for final analysis. Mean age at surgery was 21.3 ± 7.8 years. Mean follow-up time was 66.1 ± 5.5 months (range, 60–78 months). Postoperative patient-reported outcome was not associated with Beighton score, Insall–Salvati ratio, or TT–TG distance. Q angle was negatively correlated to Kujala scores and Lysholm scores. Severity of trochlear dysplasia was not associated with postoperative patient-reported outcome. The mean Kujala score increased from 69.4 ± 7.9 to 96.1 ± 1.9, the mean Tegner score increased from 3.1 ± 1.3 to 5.9 ± 1.3, and the mean Lysholm score increased from 73.5 ± 14.6 to 95.3 ± 3.4. Two patients experienced recurrent patellar dislocation during follow-up.

**Conclusions:**

MPFL reconstruction using autologous gracilis tendon under arthroscopy appears to be a reliable and safe method for treating recurrent patellar dislocation.

**Level of evidence:**

Level IV.

## Introduction

Acute patellar dislocation is a common injury, especially among adolescents, and is usually related to sports and physical activities [1]. It may be triggered by traumatic or nontraumatic events [2]. About 15–44% of patients who are treated conservatively after an acute dislocation will have recurrent patellar dislocation [3, 4]. Patients with anatomic variations such as a large Q angle, patella alta, femoral trochlear dysplasia, and arthrochalasis are particularly prone to recurrence [5–8].

There are numerous surgical options available to treat patellar dislocation but no consensus as yet on which one is the most effective. Medial patellofemoral ligament (MPFL) reconstruction can provide excellent clinical results and has been increasingly applied in recent years [9–11]. The MPFL is composed of an inferior-straight bundle at the medial aspect of the patella and a superior-oblique bundle at the superior-medial aspect of patella [12]. Because of this double-bundle structure of the MPFL, a patellar double-tunnel approach is recommended to achieve anatomical reconstruction [13, 14]. The conventional method of MPFL reconstruction usually requires two incisions: one at the patellar inner margin and another at the femoral medial epicondyle [9, 15, 16]. Because MPFL is located in the second layer, incision of the extensor apparatus is also necessary. For the patients in this study, we used endoscopic fenestration of the articular capsule to expose the medial patellar bone margin and then drilled two bone tunnels under arthroscopy to reconstruct the MPFL.

Risk factors for patellar dislocation include increased Q angle, patella alta, excessive TT–TG (tibial tubercle–trochlear groove) distance, trochlear dysplasia, and arthrochalasis. Some scholars recommend that these factors also be corrected during surgery. For example, tibial tubercle osteotomy is recommended in patients with TT–TG distance > 20 mm [17–19], as an excessively large TT–TG increases patellar lateral stress and results in lateral dislocation. Frequent dislocation leads to increased medial patellar joint pressure, which induces patellofemoral joint degeneration [20].

The purpose of this study was to evaluate outcomes in patients with recurrent patellar dislocation treated with arthroscopic MPFL reconstruction using autograft gracilis tendon and a modified double-patellar tunnel method. Patient-reported outcomes were evaluated after 5-year follow-up. We hypothesized that MPFL reconstruction using autologous gracilis tendon under arthroscopy would provide good clinical outcomes and Beighton score, Insall–Salvati ratio, TT–TG distance, and Q angle would be correlated to patient-reported clinical outcomes.

## Methods

### Patients

A total of 79 patients who underwent MPFL reconstruction using autograft gracilis tendon with modified two patellar tunnels technique between January 2012 and June 2013 were included in this retrospective study. All patients had been operated upon by the same surgeon. Patients were eligible for inclusion if (1) they had had patellar dislocation at least twice, and (2) they had been followed up for at least 5 years after the surgery. Patients with bony defects, concomitant ligament injury, or prior knee surgery were excluded from this study. The clinical, operative, and follow-up data were collected from the hospital records and analyzed retrospectively.

This study was approved by The Ethics Committee of the Third Hospital of Peking University our institutional review board (IRB 000067612014205). All participants signed informed consent before surgery.

### Functional and radiographic evaluation

Standard anterolateral radiograph of the knee in 30° flexion, Merchant-view radiographs, and knee joint CT were obtained before surgery. Preoperatively, the Beighton score and Q angle [21] were measured and recorded for all patients. The Beighton score was used to evaluate joint hypermobility by functional examination. The Insall–Salvati ratio was calculated according to the method of Insall and Salvati [22] and trochlear dysplasia was evaluated (according to the method of Dejourat et al. [17]) on a standard lateral radiograph of the knee in 30° flexion. The TT–TG distance was measured on the knee joint CT [17]. Insall–Salvati ratio, TT–TG distance, and Q angle were measured by the treating surgeon (who is also the corresponding author). The Beighton score was measured by the first author. We have planned to follow-up all patients with imaging, but only parts of patients (60.6%) were followed up with postoperative imaging because some patients refused.

### Surgery technique

**Fig. 1:**
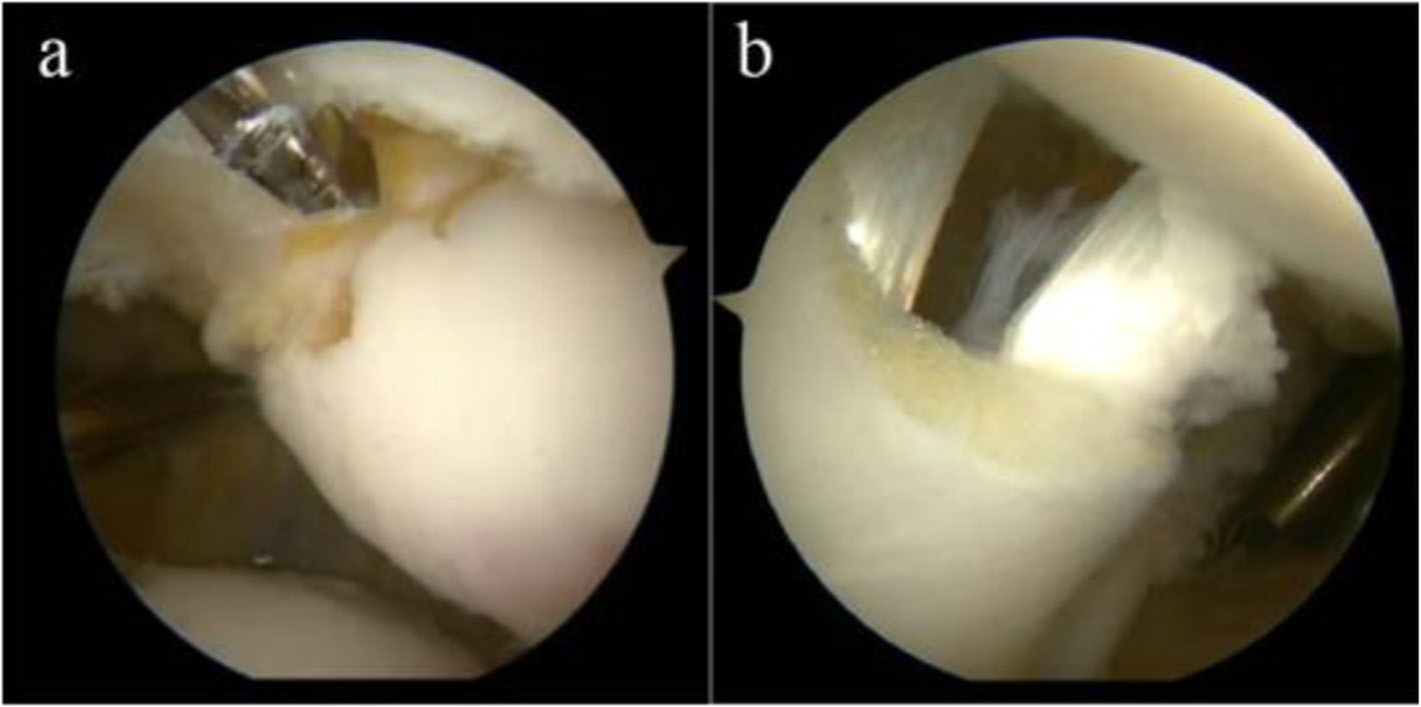
**a** Preparation of the patellar tunnel under arthroscopy. **b** Reconstructed MPFL at arthroscopy

Arthroscopy was performed first to treat any intra-articular synovitis, cartilage injury, loose bodies, and other such problems. Then, the fascia was exposed, and the gracilis tendon was harvested using a tendon stripper. The graft diameter was about 3–3.5 mm. The cut ends of the tendon were sutured together with braided suture. A capsular window was made at the medial cartilage margin of the patella under arthroscopy to expose the medial border of the patella. The patellar tunnels were prepared arthroscopically. An auxiliary approach was established about 3 cm medial to the midpoint of the inner edge of the patella as the working portal and the procedure of preparing patellar tunnel was viewed from medial arthroscopic approach. Two bone tunnels (3.5-mm diameter) were drilled at the superomedial half of the patella toward the patellar surface under arthroscopy until the cortex of patellar was penetrated. The two exits of the bone tunnels were located in the medial part of the midline of the patella surface. A distance of about 1 cm between tunnels was maintained to avoid fracturing the patella (Fig. 1a). The midpoint between the femoral medial epicondyle and the adductor tubercle was identified by palpation, and a 1-cm longitudinal incision was made. At this point, which is the anatomic termination point of the MPFL [23, 24], a femoral bone tunnel with a diameter of 5 mm was drilled under direct vision. A subcutaneous pathway was established between the longitudinal incision and the medial margin of the patella by blunt penetration. The gracilis tendon, along with a guide wire, was passed through the two patellar bone tunnels, forming U-type loop. The two ends of the tendon were drawn between the medial part of the second and third joint capsule layers to the femoral bone tunnel through the subcutaneous pathway. Then, with the knee flexed to 90°, the two ends of the graft were fixed in the femoral bone tunnel using a bioresorbable interference screw (Fig. 1b) [18, 25–27]. Finally, the surgeon confirmed under arthroscopy that the patellar tracking was appropriate and that the knee joint could be freely flexed to 110°.

### Postoperative rehabilitation

A hinged brace locked in extension was worn for 4 weeks after surgery. Partial weight-bearing exercise was started at 2 weeks after surgery and full weight-bearing exercise at 4 weeks after surgery. Range of motion exercise began 5 days after surgery, with 90° flexion being achieved by the postoperative second week. The degree of flexion was gradually increased and, on average, normal range of motion was achieved by 2 months after surgery. Controlled sports activities such as jogging were allowed at 4 months after surgery, and full return to sports was allowed at 6 months. Postoperative rehabilitation was guided by the same physical therapist.

### Outcome scores

The Kujala, Lysholm, and Tegner scores of all patients were evaluated before and after operation. Patient satisfaction with final outcome (graded as excellent, good, or fair) was documented at the end of 5 years. Complications and dislocations occurring after surgery were recorded.

### Statistical analysis

The paired *t* test (two-tailed) was used to evaluate differences between preoperative and postoperative assessments. Pearson correlation analysis was used to evaluate the relationship between postoperative patient-reported outcomes and Beighton score, Insall-Salvati ratio, Q angle, and TT–TG distance. SPSS10.0 software (SPSS Inc., Chicago, IL, USA) was used for statistical analysis. Statistical significance was at *P* < .05.

## Results

**Fig. 2:**
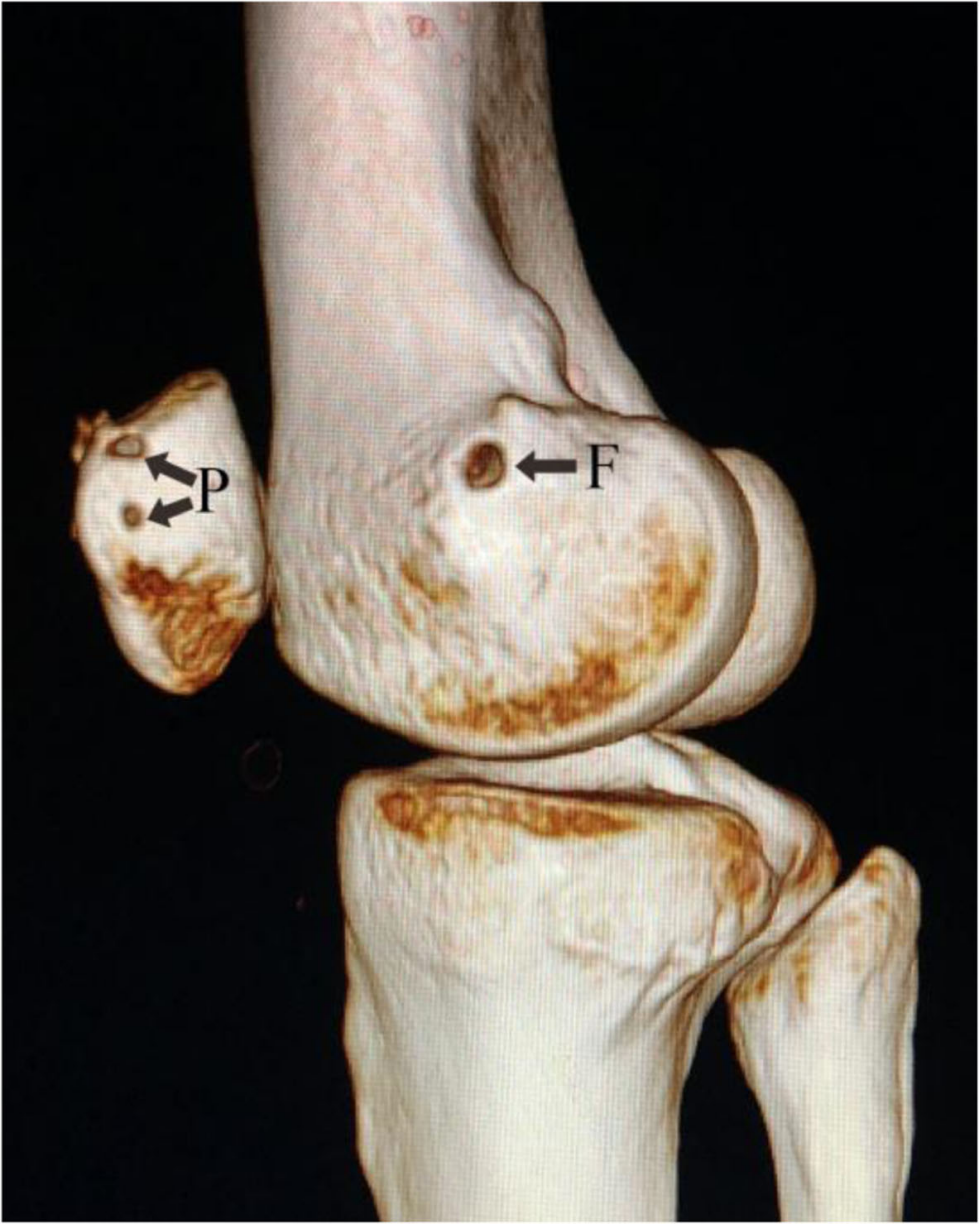
Postoperative three-dimensional CT showing the position of the bone tunnels. *F* femoral bone tunnel, *P* patellar bone tunnels

**Fig. 3:**
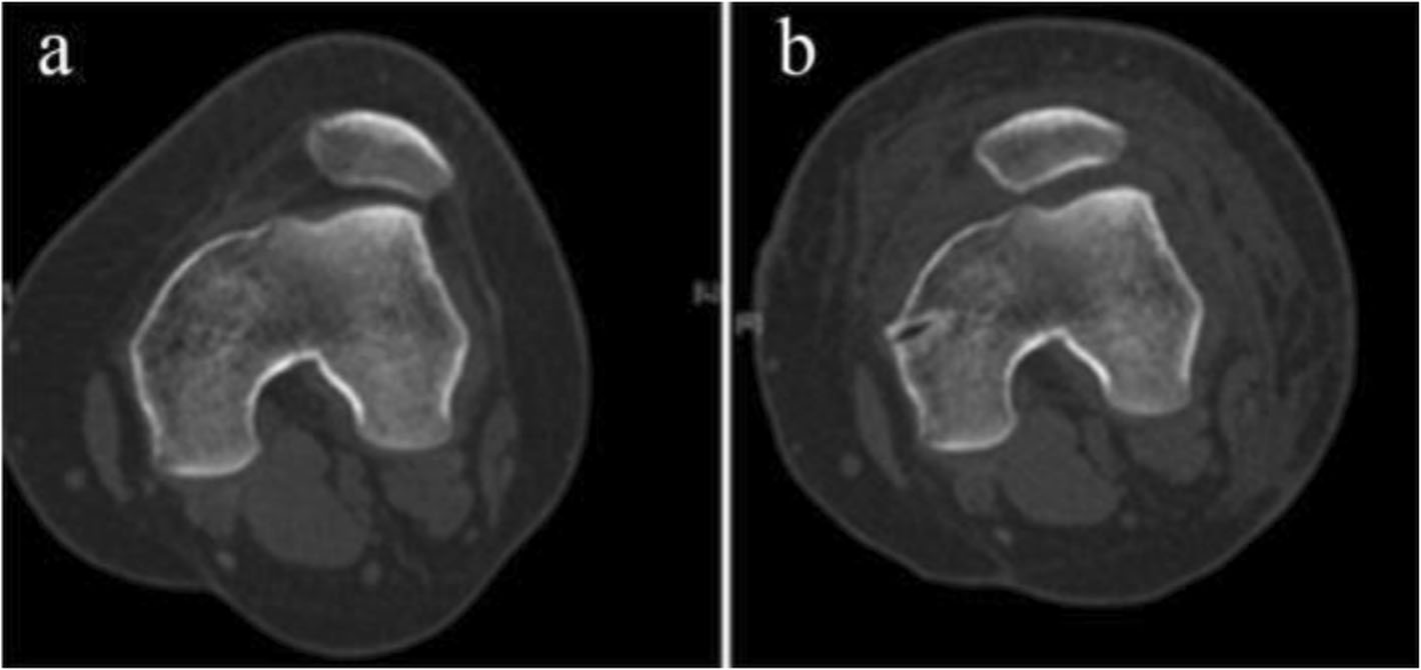
Comparison of preoperative (**a**) and postoperative (**b**) CT scans demonstrating restoration of patellofemoral tracking

**Fig. 4:**
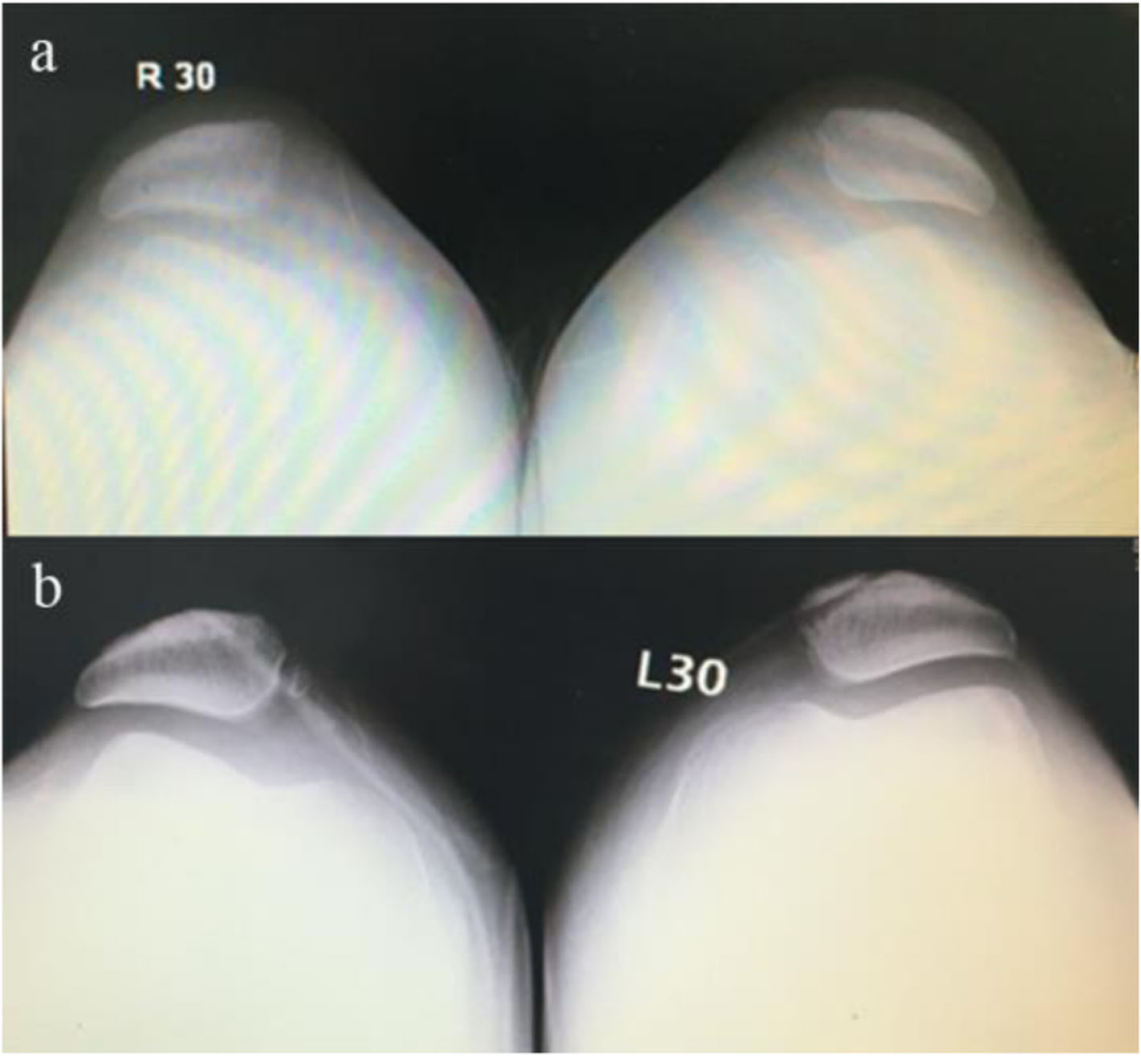
Comparison of preoperative (**a**) and postoperative (**b**) Merchant-view radiographs demonstrating restoration of patellofemoral tracking

Of the 79 patients (94 knees) initially included in the study, 13 (16.5%) patients (14/94 knees; 14.9%) were lost to follow-up. Thus, the data of 66 patients (80 knees) were available for final analysis. These 66 patients included 17 men and 49 women. Table 1 summarizes the demographic characteristics of the study patients. Mean age at the time of surgery was 21.3 ± 7.8 years (range, 13–50 years). Mean body mass index (BMI) was 22.5 (range, 18.3–27.7). Mean interval from initial injury to operation was 6.2 ± 10.1 months (range, 0.2–30 months). Mean postoperative follow-up was for 66.1 ± 5.5 months (range, 60–78 months). The clinical follow-up rate was 83.5%. Table 2 displays the results of preoperative functional and radiological evaluation. Figures 2, 3, and 4 show the postoperative CT and Merchant-view radiographs of one of our patients; there is normal congruence angle and patellar tilt angle and restoration of patellofemoral tracking. The mean Beighton score was 3.8 (range, 0–9); mean Insall–Salvati ratio was 1.3 ± 0.2 (range, 0.8–1.8); mean Q angle was 14.5 ± 3.5° (range, 5–28°); and mean TT–TG value was 15.6 ± 1.8 mm (range, 8–23 mm). As Table 3 shows, there was no relationship between postoperative patient-reported outcomes and the Beighton score, Insall–Salvati ratio, or TT–TG distance. The Q angle was negatively correlated to the Kujala score and the Lysholm score (*r* value = − 0.421, *p* = .009). Severity of trochlear dysplasia was classified according to Dejour (types A–D). Type A trochlear dysplasia was seen in 41 patients, type B in 20 patients, type C in 12 patients, and type D in 7 patients. The severity of trochlear dysplasia was not associated with postoperative patient-reported outcomes. Table 4 shows the patient-reported outcomes and satisfaction degree. At final follow-up, the mean Kujala score had improved from 69.4 ± 7.9 before surgery to 96.1 ± 1.9, the Tegner score from 3.1 ± 1.3 to 5.9 ± 1.3, and the Lysholm score from 73.5 ± 14.6 to 95.3 ± 3.4. All scores demonstrated significant improvement (*P* < .001). The outcome of the operation was graded as “excellent” by 57 (86.4%) patients, as “good” by eight (12.1%) patients, and as “fair” by one (1.5%) patient. Two patients experienced recurrent patellar dislocations. One was a young man who suffered a knee injury while exercising 8 months after surgery. He refused revision surgery after manipulative reduction. He gave up strenuous exercise and has not suffered another dislocation so far. The other patient was a young woman who sprained her knee joint and then suffered re-dislocation while going down the stairs 1 year after surgery. One month after the re-dislocation, she underwent tibial tubercle osteotomy with revision MPFL repair. One year after the revision surgery, she returned to normal activity and currently experiences only occasional pain when going up and down the stairs. No infections, effusion, or screw site pain were encountered during follow-up.

**Table 1:** Patient demographics

	Value
Number of patients	66
Number of knees	80
Follow-up rate, %	83.5
Mean age at surgery, years	21.3 ± 7.8 (13–50)
BMI, kg/m^2^	22.5 (18.3–27.7)
Duration of symptoms, months	6.2 ± 10.1 (0.2–30)
Gender (F/M), *n*	49/17
Side (L/R), *n*	40/40
Follow-up period, months	66.1 ± 5.5 (60–78)
Number of recurrent patellar dislocations	2

Values are shown as mean ± SD (range) unless otherwise indicated

**Table 2:** Preoperative functional and radiographic evaluation

	Value
Q angle	14.5 ± 3.5° (5–28°)
TT-TG distance, mm	15.6 ± 1.8 (8–23)
Insall-Salvati ratio	1.3 ± 0.2 (0.8–1.8)
Beighton score	3.8 ± 2.6 (0–9)
Dejour trochlear dysplasia, *n* (%)	
Type A	41 (51.3)
Type B	20 (25.0)
Type C	12 (15.0)
Type D	7 (8.8)

Values are shown as mean ± SD (range) unless otherwise indicated

**Table 3:** Relationship between postoperative patient-reported outcomes and Beighton score, Insall-Salvati ratio, Q angle, and TT–TG distance

	Kujala score		Tegner score		Lysholm score	
	r value	*P* value	r value	*P* value	r value	*P* value
Q-angle	− 0.421	0.009	− 0.202	0.223	− 0.413	.010
TT–TG distance	− 0.112	0.502	− 0.096	0.565	− 0.159	.342
Insall–Salvati ratio	− 0.131	0.433	− 0.107	0.523	− 0.089	.597
Beighton score	− 0.017	0.918	0.034	0.841	− 0.077	.645

**Table 4:** Patient-reported outcomes and satisfaction degree

	Preoperative	Postoperative	*P* value
Kujala score	69.4 ± 7.9	96.1 ± 1.9	*P* < .001
Tegner score	3.1 ± 1.3	5.9 ± 1.3	*P* < .001
Lysholm score	73.5 ± 14.6	95.3 ± 3.4	*P* < .001
Subjective satisfaction, *n* (%)			
Excellent		57 (86.4)	
Good		8 (12.1)	
Fair		1 (1.5)	

Values are shown as mean ± SD unless otherwise indicated

## Discussion

The mid-term clinical outcomes show that MPFL reconstruction using autologous gracilis tendon graft and the double-patellar tunnel technique under arthroscopy appears to be a reliable and safe method for treating recurrent patellar dislocation. The Kujala score, the Tegner score, and the Lysholm score demonstrated significant improvement. Postoperative patient-reported outcome was not associated with Beighton score, Insall–Salvati ratio, TT–TG distance, or severity of trochlear dysplasia. Q angle was negatively correlated to Kujala scores and Lysholm scores.

MPFL is the main structure preventing lateral dislocation of the patella [28, 29], and therefore patellar dislocation is invariably associated with MPFL rupture [30, 31]. MPFL is composed of double functional bundles, including an inferior-straight bundle at the medial aspect of the patella and a superior-oblique bundle at the superior-medial aspect of patella [12]. The patellar double-tunnel technique is recommended by many authors as the best method for achieving anatomical reconstruction [13, 32–34]. The improvement in Kujala scores are reported to be similar to that achieved with other operative techniques [32, 35, 36]. In the patellar double-tunnel technique, two parallel tunnels are drilled through the patella from the medial margin to the lateral margin of the patella. In previous studies, the diameters of the bone tunnels have ranged from 3.2 to 4.5 mm [9, 32, 37, 38]. The existence of the patellar tunnels damages patellar integrity and strength, and there are several reports of patellar fracture occurring after MPFL reconstruction [9, 39]. Some surgeons therefore avoid the patellar tunnel technique and prefer to use a beltline rivet at the patellar inner margin to fix the graft [27, 40, 41]. However, the fixation strength of the beltline rivet is reported to be less than the strength of the bone tunnel tendon loop [42]. Reconstruction using suture anchors, which is another option, could also cause patellar fracture [43]. Studies comparing the common graft fixation methods—i.e., patellar bone tunnel fixation, beltline rivet fixation, direct suture fixation, and bone tunnel fixation without penetrating through the cortex—have found that only patellar bone tunnel fixation provides strength comparable to that of the undamaged MPFL. The strengths of beltline rivet and direct suture are particularly low [42]. We prefer to use the modified double-bone tunnel method. To prevent damage to patellar integrity, we restrict the diameter of the patellar tunnels to 3.5 mm and drill the bone tunnel from the inner lower patellar margin to the lateral upper margin so that the bone tunnel outlet is at the medial half of the patellar surface. We used this technique in all patients in this study and encountered no patellar fractures over the 5-year follow-up period.

The conventional method of reconstructing the MPFL requires two incisions to be made: one at the patellar inner margin and another at the femoral medial epicondyle. The extensor apparatus is incised to reach the second joint capsule layer and expose the MPFL. This additional surgical trauma and damage to the extensor apparatus is avoided by our method, where we use endoscopic fenestration of the articular capsule to expose the medial patellar bone margin and drill the bone tunnels under arthroscopy to reconstruct the MPFL. Trochlear dysplasia was found in every patient in our study. Some researchers have performed femoral trochleoplasty to correct trochlear dysplasia and improve patellar stability. Wagner et al. [44] suggested that severe trochlear dysplasia was related to poorer outcomes following MPFL reconstruction. Kohn et al. [45] concluded that severe trochlear dysplasia was an indication for combining a trochleoplasty with MPFL reconstruction; however, their study was only a case series and did not have a comparison group. Some studies reported that combining trochleoplasty with MPFL reconstruction causes increase in patellofemoral joint pressure and leads to pain and patellofemoral joint adhesion, seriously affecting postoperative outcomes [46, 47]. However, some recent studies have shown that trochleoplasty can provide good clinical results [48, 49]. In the present study, for patients with arthrochalasis, patellar alta, trochlear dysplasia, and excessively large TT–TG, only simple MPFL reconstruction and lateral retinaculum release were performed. Although the above-mentioned high-risk anatomical factors were not corrected, postoperative outcomes were still satisfactory. In fact, we found that postoperative outcome is not affected by these high-risk factors (other than the Q angle), which is in line with earlier reports [19, 36, 50, 51].

The Q angle was found to be negatively correlated to the Kujala score and the Lysholm score in this study. A large Q angle causes greater lateralization force on the patella, which increases the retropatellar pressure between the lateral facet of the patella and the lateral femoral condyle. This may explain the negative correlation between the Q angle and patient-reported outcomes. Our operation mainly aimed to restore the anatomy of the affected knee to the status before the first dislocation and thus prevent recurrent patellar dislocation. Restoration of anatomy and reconstruction of the MPFL are probably most beneficial for patients with MPFL rupture caused by trauma and congenitally weak medial structures. However, we recognize that some patients may still be at high risk for patellar dislocation even after MPFL reconstruction.

This study has several limitations. First, this was a retrospective study with a small sample. Second, it was a single-center, single-surgeon study; the results may not reflect the experience at other centers. Third, all patients did not undergo postoperative imaging follow-up. Fourth, a control group was not included. Given the difficulty in obtaining adequate films and the variable interpretation of these images, the use of the four-grade Dejour classification to evaluate trochlear dysplasia can also be considered a limitation of this study [52, 53]. We have tried our best to obtain adequate films and maybe we can use CT or MRI to evaluate trochlear dysplasia in later studies.

## Conclusion

MPFL reconstruction using autologous gracilis tendon graft and the double patellar tunnel technique under arthroscopy appears to be a reliable and safe method for treating recurrent patellar dislocation. The mid-term clinical outcomes in our sample were good.

## Data Availability

Not applicable.

## Abbreviation

MPFL: Medial patellofemoral ligament
